# Kinetic analysis and parameter optimization of shrimp shell liquefaction

**DOI:** 10.3389/fbioe.2026.1936936

**Published:** 2026-10-20

**Authors:** Jie Zhang, Xiao-Chun Liu, Ao Chen, Hui-Yuan Zhang, Yu-Cang Zhang

**Affiliations:** 1 Guangzhou Railway Polytechnic, Guangzhou, China; 2 Hebei Key Laboratory of Hazardous Chemicals Safety and Control Technology, School of Chemical Safety, University of Emergency Management, Langfang, China; 3 College of Food and Biological Engineering, Jimei University, Xiamen, China

**Keywords:** kinetics, liquefaction, optimization, response surface method, shrimp shell, mechanism

## Abstract

This study addresses the environmental issues caused by massive global shrimp shell waste by optimizing its liquefaction conditions and analyzing liquefaction kinetics at different temperatures. Liquefaction was conducted using polyethylene glycol 400–glycerol as the mixed solvent and concentrated sulfuric acid as the catalyst. Response surface methodology (RSM) was employed to optimize reaction temperature, liquefaction time, liquid–solid ratio, and catalyst dosage. A kinetic model based on chemical reaction kinetics and transition state theory was established to evaluate the effect of temperature on the liquefaction rate. The optimal conditions were 173 °C, a liquid–solid ratio of 14:1, a catalyst dosage of 7%, and a reaction time of 108 min, achieving a liquefaction rate of 42.35% ± 0.84%. The process was confirmed as a complex multistage reaction; the apparent rate constant rose with increasing temperature, and the activation energy was 64.52 kJ/mol. Kinetic analysis showed that activation Gibbs free energy, enthalpy, and entropy varied with temperature, suggesting the formation of activated complexes. Overall, shrimp shell liquefaction is a temperature-dependent multistage process, and the kinetic activation parameters lay a theoretical basis for revealing its liquefaction mechanism.

## 1 Introduction

Approximately 6–8 million tons of shrimp and crab shell waste are generated worldwide each year. Most of this waste is simply discarded, with only a small portion used as animal feed or in other applications. This practice inefficiently uses resources and causes environmental pollution (Zhang et al., 2012; Chen and Yan, 2014; Bobbink et al., 2015; Yan and Chen, 2015). Direct abandonment of crustacean shell waste triggers dual resource and environmental risks: on land, shell degradation consumes massive soil nitrogen and triggers malodorous gas emissions (Xiao et al., 2018; Sasmoko et al., 2022; Shams et al., 2026); in offshore areas, accumulated shells cause coastal sediment siltation and disrupt benthic biological habitats. China is a major maritime nation and the world’s largest seafood producer, accounting for more than one-fifth of the global seafood production. In recent years, as the seafood processing industry has rapidly developed, the amount of shell waste has continued to increase (Gates, 2011; Pădurețu et al., 2019). Traditional low-value recycling methods cannot keep pace with the output growth, so high-value green utilization of shrimp shell waste has become a research hotspot in marine biomass resource recovery (Li et al., 2020; Wang et al., 2023; Bello et al., 2026; Manju et al., 2026).

Shrimp shells are typical composite marine biomass mainly composed of chitin (20%–30%), calcium carbonate (30%–50%), and structural proteins (15%–25%), with abundant hydroxyl, acetamido, and amino active functional groups distributed on chitin molecular chains (Arnold et al., 2021; Zhong et al., 2023; Zhao and Zhiani, 2025). Unlike rigid terrestrial lignocellulosic biomass, shrimp shell has a loose layered microstructure, making it easier to undergo solvent-induced molecular depolymerization under mild reaction conditions (Balani et al., 2011; Popov, 2014; Liang et al., 2016; Dang et al., 2019). Developing and utilizing shrimp shell waste is an effective approach for addressing environmental protection and energy-related challenges (Ahmed et al., 2025; Hu et al., 2026; Zhao et al., 2026).

Shrimp shell waste contains abundant active functional groups and can be liquefied in suitable solvents, similar to wood waste (Zhang et al., 2018; Zheng et al., 2019; Deng et al., 2020; Suryawanshi and Eswari, 2022). Liquefaction is one of the simplest and most effective techniques for converting solid biomass into liquid polyols or aromatics (Zhao et al., 2012). Typically, this process proceeds at 150 °C–170 °C under atmospheric pressure, with polyhydric alcohols such as polyethylene glycol (PEG), diethylene glycol (DEG), and ethylene glycol (EG) serving as solvents and acids acting as catalysts. Yan et al. first applied the liquefaction method to convert chitin into small-molecule chemicals such as HADP, HAADP, NAG2F, and DH1 (Pierson et al., 2014; Zhang and Yan, 2016). Following this route, our team further pioneered the application of liquefied chitin derivatives for polymer modification (Zhang et al., 2018; Zheng et al., 2019). In those works, ball-milled chitin or ball-milled shrimp shells were liquefied into polyols using a PEG 400/glycerol mixed solvent system with sulfuric acid as the catalyst. Deacetylation and depolymerization take place throughout the liquefaction process. The weight-average molecular weights (MW) of liquefied ball-milled chitin (LBMC) and liquefied ball-milled shrimp shells (LBMS) were determined to be 7,192 Da and 9,711 Da, respectively. The resulting liquefied products can be used to prepare biodegradable films, foams, and adhesives (Li et al., 2015; Yang et al., 2018; Zhang et al., 2018; Zhang et al., 2020; Chen F et al., 2023; Huang et al., 2023).

Biomass liquefaction is recognized as one of the most cost-effective and scalable conversion technologies for low-grade biomass (Huang and Yuan, 2015; Gollakota et al., 2018; Zhang et al., 2019; Bontaş et al., 2023). It relies on thermal and solvent synergistic effects to break intramolecular hydrogen bonds and covalent bonds of biomass macromolecules, converting insoluble solid biomass into solvent-soluble oligomer fragments with reactive functional groups (Yamada et al., 2007; Kunaver et al., 2010; Xu and Li, 2021; Akbarian et al., 2022; Chen Z et al., 2023). To better understand how liquefaction variables affect the liquefaction rate, the chemical kinetics of the liquefaction process must be investigated before applying the liquefied shrimp shell products as raw materials for polymer materials. Considerable progress has been made in studies on the liquefaction kinetics of wood waste. Xiao et al. (2022) investigated the chemical kinetics of corn straw in polyol solvents. Yan et al. (2010) examined the liquefaction kinetics of *Salix psammophila* in ethylene carbonate. Shi et al. (2016) studied the chemical kinetics of rubber seed shell in polyol solvents. However, the existing kinetic models cannot fully and systematically describe the dynamic characteristics of shrimp shell liquefaction under polyol solvent systems. Systematic, targeted kinetic exploration of shrimp shell liquefaction is still insufficient. This research gap restricts the mechanistic interpretation of shrimp shell liquefaction. Therefore, this study aims to fill the above gap by exploring the chemical kinetics of shrimp shell liquefaction.

Herein, shrimp shells were liquefied using polyethylene glycol 400 and glycerol as a green mixed polyol solvent. Compared with single-component polyol solvents widely adopted in earlier biomass liquefaction studies, PEG 400 possesses low volatility, appropriate viscosity, and strong hydrogen-bonding interaction with chitin macromolecules. When blended with glycerol, the binary solvent system achieves superior swelling and infiltration performance toward chitin-rich marine biomass, which helps weaken the barrier effect induced by calcium carbonate inside shrimp shells. Response surface methodology (RSM) was applied to systematically optimize four core liquefaction variables, including reaction temperature, solvent-solid ratio, reaction time, and catalyst dosage, to alleviate the inhibitory effect of calcium carbonate on solvent infiltration and improve the liquefaction efficiency of shrimp shells. Most relevant literature has focused on liquefying purified chitin; systematic optimization and kinetic analysis targeting raw shrimp shells containing calcium carbonate are still insufficient. To address this gap and using optimized experimental data, a liquefaction kinetic model adapted to shrimp shell composite components was established to calculate apparent activation energy and reaction rate constants. Unlike previous investigations that focused only on liquefaction yield or product characterization, this work further reveals the kinetic characteristics of the shrimp shell liquefaction process, providing a targeted theoretical basis for further understanding the liquefaction mechanism of shrimp shell and the large-scale industrial application of liquefaction products in biodegradable polymer fabrication.

## 2 Materials and methods

### 2.1 Materials

Shrimp shells were collected from a nearby seafood processing plant and dried to a constant weight in an oven at 105 °C. Polyethylene glycol, glycerol, 95% ethanol, and concentrated sulfuric acid (98%) were of analytical grade. A Pulverisette 6 ball mill (Fritsch, Germany) and a Value HL G2 rotary evaporator (Heidolph, Germany) were used.

### 2.2 Pretreatment of shrimp shell

The dried shrimp shells were crushed to 60–80 mesh and dried again in an oven at 105 °C to constant weight. The shrimp shell powder was further pretreated by ball milling (Zhang and Yan, 2016). For ball milling, specified amounts of shrimp shell powder and zirconia balls (3 mm) were placed in the ball mill tank and operated at 500 rpm/min for 30 min, followed by standing for 60 min, repeated for eight cycles. The treated powder was stored in a sealed container.

### 2.3 Liquefaction of shrimp shell

A specified amount of shrimp shell powder and liquefying agent (polyethylene glycol: glycerol = 4:1, *w/w*) was added to a three-necked flask, magnetically stirred, condensed, and refluxed. The mixture was heated in an oil bath to the set temperature and allowed to stabilize. The catalyst (concentrated sulfuric acid) was then added. When the preset reaction time was reached, the flask was placed in an ice bath to terminate the reaction. The solid residues were separated by vacuum filtration using a Buchner funnel and repeatedly washed with ethanol until the filtrate became colorless. The obtained residues were dried in an oven at 105 °C to constant weight.

### 2.4 Analytical methods

#### 2.4.1 Determination of liquefaction rate of liquefaction products

The product was dissolved in 95% ethanol and vacuum filtered. The residue was washed with 95% ethanol until the filtrate became colorless. The residue was dried in an oven at 105 °C to constant weight, cooled to room temperature in a desiccator, and weighed. Referring to the method reported by Zheng et al. (2019), the liquefaction rate, *Y*, was calculated as follows:

$$Y=\frac{W_{0}-W}{W_{0}}\times 100\%,$$ (1)

where *Y* (%) is the liquefaction rate of shrimp shell, *W*_0_ (g) is the initial mass of shrimp shell powder, and *W* (g) is the residue mass at time *t*. It should be noted that the dissolution of CaCO_3_ is limited under the liquefaction conditions adopted in this work. Therefore, the calculated liquefaction rate mainly reflects the conversion level of organic biomass.

#### 2.4.2 Experimental design

All experiments in this study were performed in triplicate. Based on single-factor experiments, a four-factor three-level Box–Behnken design was adopted using Design-Expert software (Wang et al., 2022). Reaction temperature (*X*_1_), liquefaction time (*X*_2_), liquid–solid ratio (*X*_3_), and catalyst dosage (*X*_4_) were selected as independent variables, and the liquefaction rate (*Y*) of shrimp shell powder was used as the response variable. The response surface methodology was applied to optimize the liquefaction process of shrimp shell powder, as shown in Table 1.

**TABLE 1:** Factors and levels of test design.

Factor	Level		
	−1	0	1
*X*_*1*_	160	170	180
*X*_*2*_	80	110	140
*X*_*3*_	12	14	16
*X*_*4*_	6	7	8

#### 2.4.3 Kinetic model

Owing to the excessive dosage of liquefying agent relative to shrimp shell feedstock, the concentration of liquefying agent in the reaction system remains nearly constant throughout the reaction. In addition, the catalyst dosage is fixed for a given reaction. Therefore, the reaction rate can be assumed to depend only on the liquefaction rate (Zhang et al., 2014). To further simplify the study of the liquefaction chemical kinetics of shrimp shells, the following four assumptions were made (Yan et al., 2010): (a) the concentration of the liquefying agent remains constant throughout the reaction; (b) the concentration of the catalyst remains constant throughout the reaction; (c) the variation of the reaction rate constant with temperature (T) follows the Arrhenius equation; and (d) the reaction is irreversible. The liquefaction process of shrimp shell in a mixed polyol solvent can be regarded as an irreversible bimolecular reaction.

$$x+y↔xy^{*}\to \text{Products}.$$ (2)

The chemical kinetics model is expressed as follows:

$$\frac{dy}{dt}=-kx^{n_{1}}y^{n_{2}},$$ (3)

where *x* and *y*, respectively, represent the polyhydric alcohol reagents and shrimp shell powders; *t* is the reaction time (min); *n* is the apparent reaction order; and *k* is the apparent reaction rate constant, which is associated with the Arrhenius equation:

$$k=A\exp^{-E/RT},$$ (4)

where A is the pre-exponential factor (s^−1^) and *E* is the activation energy (kJ/mol).

As the solvent is in excess, the concentration of liquefaction solvent is commonly considered a constant, and the reaction rate is only dependent on the solid residue content. Thus, the kinetic equation can be expressed as follows:

$$-\frac{dy}{dt}=ky^{n}.$$ (5)

In association with Equation 1, the logarithmic form of the kinetic equation above is

$$\ln\left(-\frac{dW}{W_{0}dt}\right)=lnk+nln\left(\frac{W}{W_{0}}\right).$$ (6)

The plot of ln (−*dW*/(*W*_0_ *dt*)) against ln (*W*/*W*_0_) is a straight line. *n* can be determined from the slope of the plot, and *k* can be calculated from the intercept.

Considering Equation 4, the dependence of the rate constant on temperature can be defined by the following equation:

$$\ln k=\text{lnA}-\frac{E}{RT},$$ (7)

where *T* is the temperature (K) and *R* is the gas constant (8.314 J/(molK)). The plot of ln *k* versus 1/*T* is a straight line, and its slope and intercept can be used to determine *E* and A, respectively.

A more detailed explanation with regard to the transformation of reactants into liquefied products can be described by the transition state theory (Acemioğlu and Mehmet, 2002). In Equation 2, xy* is the intermediate state, which can be determined by the equilibrium constant. Then the activation free enthalpy (Δ*G*) of the reaction can be estimated using the Eyring equation:

$$k=\frac{k_{B}T}{h}\cdot e^{-\frac{\Delta G}{RT}},$$ (8)

where *k*_B_ is the Boltzmann constant (1.381 × 10^−23^ J/K) and *h* is the Planck constant (6.626 × 10^−34^ J·s).

Thus, the apparent activation free enthalpy (Δ*G*) can be expressed as

$$\Delta G=RT\left(\ln\left(\frac{k_{B}T}{h}\right)-lnk\right).$$ (9)

Moreover, the apparent activation enthalpy (Δ*H*) and apparent activation entropy (Δ*S*) can be calculated from Equations 10, 11, respectively:

ΔH=E−RT (10)

and

$$\Delta S=\frac{\Delta H-\Delta G}{T}.$$ (11)

## 3 Results and discussion

### 3.1 Establishment of response surface mathematical model

The detailed experimental design scheme and corresponding test results of response surface analysis for shrimp shell powder liquefaction reaction are summarized in Table 2. A four-factor three-level Box–Behnken design was adopted in this work. Runs 1–24 correspond to the axial edge points of the experimental domain, covering the variation range of every single factor and the interactive combinations of different variables. These experimental points can effectively reflect the liquefaction response characteristics of shrimp shell powder under diverse variable conditions and lay a data foundation for subsequent regression model construction. Experiments 25–29 were designed as center-point tests of the response surface model. The center-point tests were repeatedly carried out at the central level of all independent variable design ranges, aiming to verify the fitting reliability and stability of the regression equation at the core position of the experimental design space and eliminate systematic errors caused by experimental operation and environmental fluctuation to ensure the rationality of the overall experimental design.

**TABLE 2:** Test design and results of response surface analysis.

Number of tests	*X*_*1*_	*X*_*2*_	*X*_*3*_	*X*_*4*_	*Y/%*
1	−1	−1	0	0	38.79
2	1	−1	0	0	40.97
3	−1	1	0	0	39.19
4	1	1	0	0	41.21
5	0	0	−1	−1	39.15
6	0	0	1	−1	40.27
7	0	0	−1	1	40.75
8	0	0	1	1	41.85
9	−1	0	0	−1	37.54
10	1	0	0	−1	39.81
11	−1	0	0	1	40.70
12	1	0	0	1	42.14
13	0	−1	−1	0	40.78
14	0	1	−1	0	41.55
15	0	−1	1	0	41.72
16	0	1	1	0	41.68
17	−1	0	−1	0	39.10
18	1	0	−1	0	40.40
19	−1	0	1	0	39.12
20	1	0	1	0	41.82
21	0	−1	0	−1	39.17
22	0	1	0	−1	40.11
23	0	−1	0	1	41.82
24	0	1	0	1	41.14
25	0	0	0	0	42.57
26	0	0	0	0	42.03
27	0	0	0	0	43.15
28	0	0	0	0	42.76
29	0	0	0	0	42.85

Based on the above standardized experimental design and operational procedures, the core evaluation index of this study, namely, the liquefaction rate (*Y*) of shrimp shell powder corresponding to each group of response surface tests, was accurately calculated and obtained through liquefaction reaction experiments and data statistical processing. To further clarify the quantitative relationship between each independent variable and the liquefaction rate response value and screen the significant influencing factors of the liquefaction reaction, the quadratic polynomial regression fitting was performed on the liquefaction rate (*Y*) and the corresponding multi-dimensional variable data. The analysis of variance (ANOVA) of the established regression model was completed on this basis, and the detailed ANOVA results, including model significance, factor interaction significance, and fitting error analysis, are presented in Table 3, which provides a reliable statistical basis for verifying the validity of the regression model and determining the optimal process parameters for shrimp shell powder liquefaction.

**TABLE 3:** Regression equation.

Source	Sum of squares	*df*	Mean square	*F* value	*p-*value
Model	53.22	14	3.80	19.76	<0.0001**
*X*_*1*_	11.82	1	11.82	61.44	<0.0001**
*X*_*2*_	0.22	1	0.22	1.15	0.3015
*X*_*3*_	1.86	1	1.86	9.69	0.0076*
*X*_*4*_	12.71	1	12.71	66.07	<0.0001**
*X*_*1*_ *X*_*2*_	0.01	1	0.01	0.03	0.8579
*X*_*1*_ *X*_*3*_	0.49	1	0.49	2.55	0.1328
*X*_*1*_ *X*_*4*_	0.17	1	0.17	0.90	0.3601
*X*_*2*_ *X*_*3*_	0.16	1	0.16	0.85	0.3715
*X*_*2*_ *X*_*4*_	0.66	1	0.66	3.41	0.0860
*X*_*3*_ *X*_*4*_	0.0001	1	0.0001	0.0005	0.9821
*X*_1_^2^	18.45	1	18.45	95.89	<0.0001**
*X*_2_^2^	3.84	1	3.84	19.93	0.0005*
*X*_3_^2^	3.76	1	3.76	19.55	0.0006*
*X*_4_^2^	9.80	1	9.80	50.92	<0.0001**
Residual	2.69	14	0.19	​	​
Lack of fit	2.00	10	0.20	1.16	0.4811
Pure error	0.69	4	0.17	​	​
Cor total	55.92	28	​	​	​
R	0.9518	​	​	​	​

* is significant, ** is highly significant.

### 3.2 Analysis of response surface optimization

According to the experimental design, a regression equation was established with *X*_1_, *X*_2_, *X*_3_, and *X*_4_ as independent variables and *Y* as the response variable:

$$Y=-581.27+5.75X_{1}+0.36X_{2}+2.94X_{3}+23.28X_{4}-0.0001X_{1}X_{2}+0.0175X_{1}X_{3}-0.0208X_{1}X_{4}-0.0034X_{2}X_{3}-0.0135X_{2}X_{4}-0.0025X_{3}X_{4}-0.0169X_{1}^{2}-0.0009X_{2}^{2}-0.1904X_{3}^{2}-1.2289X_{4}^{2}.$$ (12)

Further statistical analysis used the ANOVA data in Table 3 to elucidate the internal correlation between various process variables and liquefaction performance. The results showed no straightforward linear correlation between any single experimental factor and the liquefaction rate of shrimp shell powder within the investigated variable ranges, suggesting that the liquefaction reaction is governed by complex multivariate coupling effects rather than simple linear variation. The quadratic polynomial regression model established in this work showed strong statistical significance, with an extremely low *p*-value of less than 0.0001 and a high F-value of 19.76, confirming the outstanding explanatory ability and statistical credibility of the constructed model. Correspondingly, the lack-of-fit test result showed no significant difference (P = 0.4811 > 0.05), indicating that the deviation between model-predicted values and actual experimental data was negligible, and the regression model could accurately and adequately fit the measured experimental data of shrimp shell powder liquefaction. The model summary statistics are as follows: R^2^ = 0.9518, adjusted R^2^ = 0.9037, predicted R^2^ = 0.7744, CV = 1.07%, and adequate precision = 16.31. The coefficient of determination (R^2^) of 0.9518 indicates that more than 95% of the variation in the shrimp shell liquefaction rate is explained by the established quadratic regression model, demonstrating high fitting precision and satisfactory applicability. The predicted R^2^ is reasonably consistent with the adjusted R^2^, and their difference is less than 0.2. Adequate precision represents the signal-to-noise ratio; a value above 4 is desirable. The adequate precision of 16.31 confirms sufficient signal, meaning the model is suitable to navigate the design space.

Significance analysis of individual variables revealed distinct influences of different process parameters on the liquefaction rate. Specifically, independent variables *X*_*1*_ and *X*_*4*_ had highly significant effects on the liquefaction efficiency of shrimp shell powder, with their corresponding P-values below 0.001. In contrast, variable *X*_*3*_ showed a relatively moderate but statistically significant influence on the liquefaction rate (P < 0.05), while variable *X*_*2*_ had no remarkable significant effect within the experimental range. By comparing the significance level and influence degree of each factor, the dominant order of the parameters affecting the liquefaction rate of shrimp shell powder was determined to be *X*_*4*_ *> X*_*1*_ *> X*_*3*_ *> X*_*2*_, providing a targeted theoretical reference for the subsequent optimization of liquefaction process conditions.

Figure 1 presents three-dimensional response surface plots illustrating the effects of interactions among the liquefaction conditions on the liquefaction rate. The results obtained from the optimal response surface values and slope analyses are consistent with those shown in Table 3. The order of influence of *X*_1_, *X*_2_, *X*_3_, and *X*_4_ on the liquefaction rate is as follows: *X*_4_ > *X*_1_ > *X*_3_ > *X*_2_. Figures 1a,d,e show that the liquefaction rate initially increases and then decreases with increasing reaction time. Figures 1a–c indicate that the liquefaction rate first increases and then decreases with increasing reaction temperature. Figures 1c,e,f demonstrate that the liquefaction rate increases markedly with increasing catalyst dosage. The liquefaction rate of shrimp shell does not exceed 43.09% even when temperature and catalyst dosage are increased simultaneously, indicating that excessively high or low values of the independent variables can hinder the liquefaction process.

**FIGURE 1:**
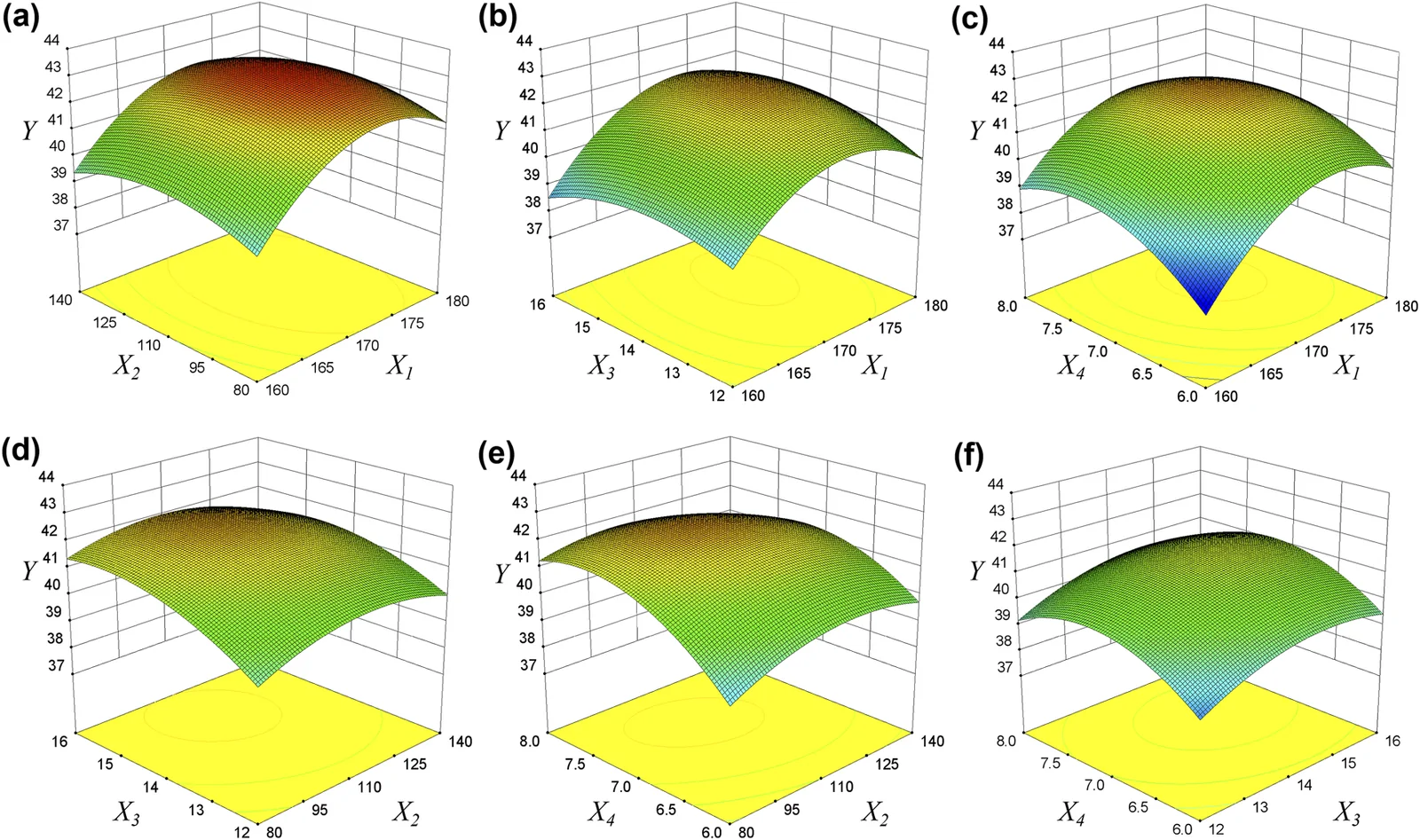
Effects of reaction temperature (X1), liquefaction time (X2), liquid–solid ratio (X3), and catalyst dosage (X4) on the liquefaction rate (Y) of shrimp shell. **(a–f)** Response surface plots showing pairwise interaction effects between the variables.

Based on the established and verified quadratic regression model, the response surface methodology (RSM) was further adopted to determine the optimal process parameters for the shrimp shell powder liquefaction reaction. Through model simulation and iterative optimization calculations, the model predicted the following theoretical optimal liquefaction conditions: a reaction temperature of 173.05 °C, a liquid-solid ratio of 14.67:1, a catalyst dosage of 7.40%, and a sustained reaction time of 107.90 min. Given the inherent limitations of experimental operation, including instrument precision, the controllability of reagent proportioning, and the feasibility of process parameter adjustment, the theoretically derived parameters were rounded and optimized to obtain practical and feasible experimental conditions. The adjusted operational parameters were set as a reaction temperature of 173 °C, a liquid-solid ratio of 14:1, a catalyst dosage of 7%, and a reaction time of 108 min, which are convenient for accurate manual control and repeated experimental verification.

Three groups of parallel validation experiments were carried out under the adjusted practical process parameters to verify the accuracy and practicality of the optimized conditions obtained by response surface analysis. The experimental results showed that the actual liquefaction rate of shrimp shell powder reached 42.35% ± 0.84% under the optimized operating conditions, which was highly consistent with the model-predicted optimal liquefaction rate of 43.09%. The relative prediction error is approximately 1.72%. The small relative error between the experimentally measured value and the model-predicted value is within the acceptable range for statistical experiments. These verification results fully demonstrate that the RSM-optimized process parameters are accurate, stable, and reliable (Kunamneni and Singh, 2005). It also proves that the constructed regression model can effectively predict the liquefaction effect of shrimp shell powder under different process conditions, which has important guiding significance for the practical production and process improvement of shrimp shell liquefaction.

### 3.3 Liquefaction kinetics of shrimp shell powder

Figure 2 presents the variation of shrimp shell liquefaction rate with reaction time under different reaction temperatures, where the liquid–solid ratio and catalyst dosage were fixed at the optimal conditions of 14:1% and 7%, respectively. As shown in the figure, reaction time strongly influences the shrimp shell liquefaction efficiency. In the initial stage of the reaction, the liquefaction rate rises sharply with increasing reaction time, attributed to sufficient contact among the shrimp shell raw material, catalyst, and solvent early in the reaction stage. As the reaction proceeds, the effective reactive components in the shrimp shell are gradually consumed, and the reaction rate slows, causing the liquefaction rate to increase slowly and eventually reach a plateau. In addition, temperature positively affects the overall liquefaction performance. Under the same reaction time condition, higher reaction temperature consistently corresponds to a higher liquefaction rate, indicating that elevated temperature can effectively drive the progression of the shrimp shell liquefaction reaction throughout the investigated time range.

**FIGURE 2:**
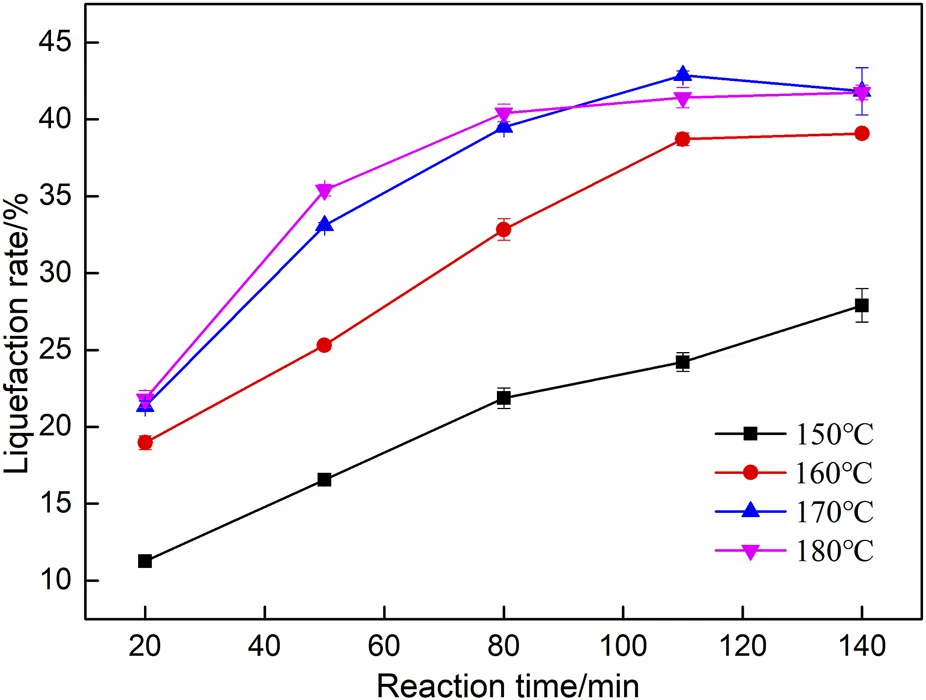
Relationship between reaction time and liquefaction rate at different temperatures.

To further explore the kinetic mechanism of the shrimp shell liquefaction process, the logarithmic relationship between liquefaction rate and reaction rate was established based on the derived Equation 6, and the corresponding fitting results are displayed in Figure 3. In accordance with the theoretical assumptions and the mathematical derivation of Equation 6, the logarithmic value of the apparent rate constant (ln *k*) is only determined by reaction temperature under constant temperature conditions and remains independent of reaction time. The fitting results further confirm that the logarithmic reaction rate shows a strong linear relationship with the logarithmic liquefaction rate as the liquefaction time increases. Using linear regression of the kinetic data at different temperatures, the key kinetic parameters of the liquefaction reaction were calculated and statistically summarized in Table 4. The table clearly shows that the apparent reaction rate constant increases monotonically with rising reaction temperature. This phenomenon confirms that high temperature positively promotes the shrimp shell liquefaction reaction, which can be reasonably explained by the fact that increased temperature elevates the internal molecular energy of chitin and other macromolecular components in shrimp shell, enabling more reactant molecules to cross the reaction activation energy barrier and thereby accelerating the bond breaking and liquefaction conversion of macromolecular structures. Moreover, the apparent reaction order of the liquefaction reaction fluctuates between 3 and 5 across different temperature conditions. This wide-range variation in reaction order shows that shrimp shell liquefaction in polyol solvent systems is not a simple elementary reaction but a complex multi-step continuous reaction involving multiple structural transformations and intermediate reaction stages.

**FIGURE 3:**
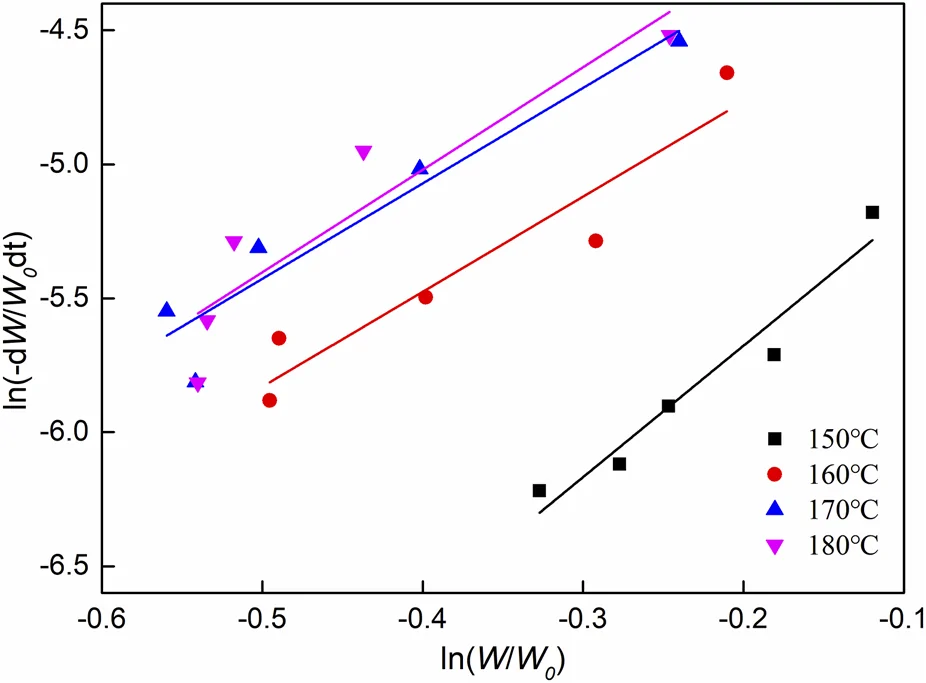
Relationship between the logarithm of the liquefaction rate and reaction rate.

**TABLE 4:** Apparent reaction rate constants and reaction orders at different temperatures.

*T*/°C	Linear equation	Apparent reaction rate constant	Apparent reaction series	*R* ^2^
150	*y* = 4.9046*x* − 4.6966	0.0091	4.90	0.9442
160	*y* = 3.5475*x* − 4.0565	0.0173	3.55	0.9029
170	*y* = 3.5599*x* − 3.6472	0.0261	3.56	0.9117
180	*y* = 3.8237*x* − 3.4909	0.0305	3.82	0.8533

To quantitatively analyze the kinetic characteristics of the shrimp shell liquefaction reaction, the kinetic parameter data of apparent rate constants at different temperatures listed in Table 4 were substituted into the established thermodynamic calculation formula (Equation 7) for further regression analysis. Subsequently, linear fitting was performed on the reciprocal of absolute temperature (1/*T*) and the natural logarithm of the apparent rate constant (ln *k*) according to the Arrhenius kinetic principle. The optimal linear fitting equation obtained in this study was determined to be *y* = −7.7606*x* + 13.751, and the intuitive fitting result is presented in Figure 4. The correlation coefficient of the fitted linear equation is 0.9708, indicating a high-degree linear correlation between the two variables. The excellent linear fit further supports the rationality of the kinetic parameter acquisition method and the reliability of the overall experimental design system for shrimp shell liquefaction. Combined with the linear fitting principle of the Arrhenius equation, the activation energy of the shrimp shell liquefaction reaction was accurately calculated based on the slope parameter of the fitted straight line, and the final activation energy was calculated from the slope of the fitted line as E = 64.52 kJ/mol. This key thermodynamic parameter provides essential numerical support for revealing the energy barrier characteristics and reaction difficulty of the shrimp shell polyol liquefaction process.

**FIGURE 4:**
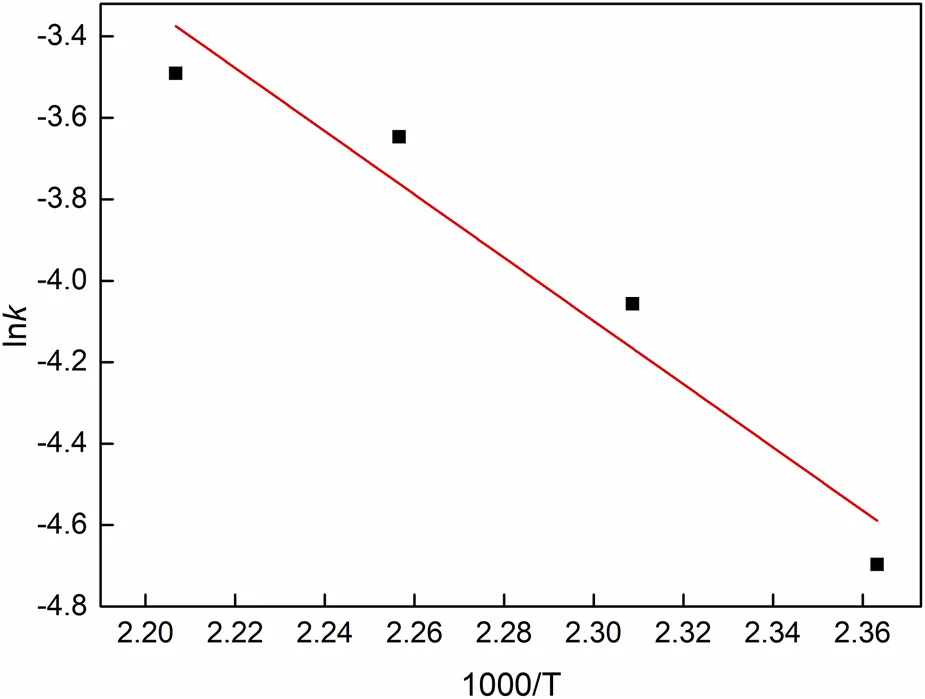
Relationship between apparent reaction rate constant and temperature.

### 3.4 Calculation of activation enthalpy and activation entropy

Although shrimp shell liquefaction is a complex multistage reaction, the overall reaction process can be interpreted using transition state theory. After the reaction begins, the reactant shrimp shell requires sufficient energy for effective collisions to form an unstable intermediate called the activated complex. As the reaction proceeds, the activated complex is transformed into liquefaction products. Activated complexes form and disappear rapidly, allowing equilibrium to be reached quickly and maintained until the end of the reaction. To further quantify the formation probability of the transition state intermediate and clarify the internal correlation between the reaction rate constant and thermodynamic parameters such as activation enthalpy, the Eyring equation was introduced for kinetic analysis in this study. This classical theoretical equation can describe the microscopic mechanism of transition-state formation in complex chemical reactions and establishes a quantitative mathematical relationship between macroscopic reaction kinetics and microscopic thermodynamic parameters.

Based on the previously determined activation energy value (*E* = 64.52 kJ/mol), the kinetic activation parameters, including Gibbs free energy of activation (Δ*G*), activation enthalpy (Δ*H*), and activation entropy (Δ*S*), at different reaction temperatures, were quantitatively calculated by substituting the kinetic parameter into the derived Equations 10, 11. The detailed thermodynamic calculation results at various temperatures are summarized in Table 5. All Δ*G* values obtained within the experimental temperature range are positive. This indicates that the reactants must overcome a certain energy barrier to form transition-state intermediates during liquefaction. Notably, Δ*G* is a kinetic parameter describing the energy barrier for reaching the transition state and cannot be used to judge the thermodynamic spontaneity of the overall liquefaction reaction. Moreover, Δ*G* shows a slow upward trend with increasing reaction temperature, and the average activation Gibbs free energy over the temperature range is calculated to be 123.14 kJ/mol. This relatively high Δ*G* further suggests that the activated complex formed in the liquefaction system is a highly unstable intermediate structure. Therefore, the system requires sufficient external thermal energy to surmount the energy barrier and sustain the liquefaction conversion.

**TABLE 5:** Δ*G*, Δ*H*, and Δ*S* at different reaction temperatures.

*T*/°C	Δ*G*/(kJ/mol)	Δ*H*/(kJ/mol)	Δ*S*/(J/(mol·K))
150	121.39	30.98	−213.65
160	122.04	30.90	−210.41
170	123.43	30.82	−208.99
180	125.71	30.73	−209.60

Further statistical analysis of activation enthalpy and activation entropy revealed the intrinsic kinetic characteristics of the liquefaction reaction. The average activation enthalpy of the shrimp shell liquefaction reaction was 30.85 kJ/mol, which was significantly lower than the typical chemical bond energy of biological macromolecular structures. This difference indicates that shrimp shell liquefaction is not a simple single bond-breaking reaction but a synergistic process accompanied by simultaneous bond cleavage and new bond formation. During the liquefaction transformation of chitin macromolecules, the original chemical bonds in shrimp shells are continuously broken, while partial polyol solvent groups are synchronously combined with degraded small-molecule liquefied products. The energy released from bond formation can partially offset the energy required to fracture the original macromolecular bonds, which is consistent with the research conclusions reported by Reshad et al. (2018). In addition, Δ*H* gradually decreases as temperature rises, demonstrating that the energy barrier for forming transition-state intermediates decreases at higher temperatures. The variation in activation enthalpy also regulates the apparent reaction order of the liquefaction process. Meanwhile, the average activation entropy of the reaction system was −211.66 J/(mol·K). The negative entropy value indicates that the degree of disorder of the reaction system decreases temporarily during the formation of the transition state, corresponding to an enhancement of system order. This phenomenon can be reasonably attributed to the solvation effect of excessive polyol solvents, which act as solvating agents to encapsulate and stabilize the intermediate products generated in the liquefaction reaction, thereby improving the thermodynamic stability of the reaction system.

## 4 Conclusion

The appropriate liquefaction conditions for shrimp shell using a mixed polyol solvent and concentrated sulfuric acid as the catalyst were determined as follows: a reaction temperature of 173 °C, liquid–solid ratio of 14:1, catalyst dosage of 7%, and reaction time of 108 min, resulting in a liquefaction rate of 42.35% ± 0.84%. The liquefaction kinetics of shrimp shell were investigated at different temperatures. The liquefaction reaction was identified as a complex multistage process that can be promoted by increasing the reaction temperature within an appropriate temperature range. Based on the Arrhenius equation and established kinetic model, the activation energy was calculated as *E* = 64.52 kJ/mol. Activated complexes formed throughout the reaction process, and Δ*G*, Δ*H*, and Δ*S* varied with increasing temperature. Excess polyols played a solvating role during the liquefaction reaction.

## Data Availability

The raw data supporting the conclusions of this article will be made available by the authors, without undue reservation.
